# Fault Diagnosis of Planetary Gearboxes Based on LSTM Improved via Feature Extraction Using VMD, Fusion Entropy, and Random Forest

**DOI:** 10.3390/e27090956

**Published:** 2025-09-14

**Authors:** Xin Xia, Haoyu Sun, Aiguo Wang

**Affiliations:** 1 School of Mechanical and Electrical Engineering, Suqian University, Suqian 223800, China; 2 Information Construction Center, Suqian University, Suqian 223800, China; sunhy@squ.edu.cn (H.S.); wangaiguo@squ.edu.cn (A.W.)

**Keywords:** fault diagnosis, planetary gearbox, fusion entropy, random forest, LSTM

## Abstract

Extracting effective fault features from the complex vibration signals of planetary gearboxes is the key to conducting efficient fault diagnosis, and it involves signal processing, feature extraction, and feature selection. In this paper, a novel feature extraction method is proposed using variational mode decomposition (VMD), fusion entropy, and random forest (RF). Firstly, VMD is employed to process the nonlinear and non-stationary signals of planetary gearboxes, which can effectively address the issues of signal modulation and mode mixing. Additionally, a fusion entropy that incorporates various refined composite multi-scale entropies is proposed; it fully utilizes the signal characteristics reflected by various entropies as features for fault diagnosis. Then, RF is adopted to calculate the importance of each feature, and appropriate features are selected to form a fault diagnosis vector, aiming to solve the problems of feature redundancy and interference in fusion entropy. Finally, long short-term memory (LSTM) is used for fault classification. The experimental results demonstrate that the proposed fusion entropy achieves higher accuracy compared with a single entropy value. The RF-based feature selection can also reduce interference and improve diagnostic efficiency. The proposed fault diagnosis method exhibits high fault diagnosis accuracy under different rotational speeds and environmental noise conditions.

## 1. Introduction

Planetary gearboxes are characterized by a compact structure, a large transmission ratio, and stable power transmission. They are widely used in mechanical transmission systems for both high-speed, high-power and low-speed, high-torque operations [1]. However, they are often subjected to complex variable loads, which can occasionally induce faults. And among these, gear faults are the primary cause of planetary gearbox mechanical failures [2]. To prevent the occurrence of such faults and thereby avoid more extensive failure cascades, many studies have been conducted on planetary gearbox fault diagnosis [3].

Most current fault diagnosis methods for planetary gearboxes rely on vibration data [4] and fall into two main categories: feature extraction combined with machine learning classification [5,6] and deep learning methods [7]. These deep learning methods require large amounts of high-quality data samples [8]. However, due to equipment privacy and data security concerns, operational data often remains in information silos, which makes it hard to obtain enough valid samples in practice. To tackle this problem, some studies have used approaches like domain generalization, federated learning, and transfer diagnosis [9,10,11] to resolve data scarcity and information silo issues. Since the planetary gearboxes have complex structures and diverse models, the applicability and generalization of these approaches still need further improvement [12]. Therefore, feature extraction combined with machine learning classification approaches is better suited to the practical needs of planetary gearboxes.

Signal processing and feature extraction serve as the foundation for fault diagnosis. A planetary gearbox comprises multiple transmission components such as the sun gear, planet gears, and ring gear. During operation, the meshing transmission between gears results in a complex vibration signal transmission path, which easily leads to signal modulation and modal aliasing [13]. Consequently, the extraction of fault response features in planetary gearboxes becomes challenging. Time frequency analysis methods can effectively address the issues of signal modulation and modal aliasing, such as wavelet transform (WT) [14], empirical mode decomposition (EMD) [15], and variational mode decomposition (VMD) [16]. VMD can effectively solve the issues of modal aliasing and endpoint effects, and it has strong anti-noise capability, making it well-suited for analyzing nonlinear and non-stationary signals [17].

Equipment faults directly cause abnormal changes in the regularity, complexity, and energy distribution of vibration signals. As a tool for quantifying the uncertainty and complexity of signals, entropy can accurately capture such abnormalities, making it serve as a key feature for fault diagnosis [18]. To capture detailed signal features across multiple scales and improve the stability of entropy calculations, researchers have proposed several refined composite multi-scale entropies, such as refined composite multi-scale sample entropy (RCMSE) [19], refined composite multi-scale dispersion entropy (RCMDE) [20], refined composite multi-scale fuzzy entropy (RCMFE) [21], and refined composite multi-scale fuzzy dispersion entropy (RCMFDE) [22]. In the process of feature extraction based on multi-scale entropy, there exists a large number of redundant features and interfering noises [23]. So, feature selection also plays a crucial role in the efficiency of fault diagnosis. Feature selection methods mainly utilize the importance or correlation of features to select features; for example, the Pearson correlation coefficient [24], mutual information (MI) [25], and importance based on random forest (RF) [26]. Feature selection based on the RF model is characterized by fast training speed and strong robustness and has been widely used in many fields [27].

In this paper, VMD is employed to process the nonlinear and non-stationary signals of planetary gearboxes, which can effectively address the issues of signal modulation and modal aliasing. A fusion entropy that incorporates various refined composite multi-scale entropies is proposed, and it makes full use of the signal characteristics reflected by each type of entropy as features for fault diagnosis. To solve the problems of feature redundancy and interference in fusion entropy, RF is adopted to calculate the importance of each feature, and appropriate features are selected to form the fault diagnosis vector so as to improve diagnostic efficiency. Finally, long short-term memory (LSTM) [28] is used for fault classification. Overall, this paper proposes a novel fault diagnosis method for planetary gearboxes based on LSTM, with improvements in feature extraction using VMD, fusion entropy, and RF. The experimental results demonstrate that the feature extraction method proposed in this paper can effectively enhance the efficiency of fault diagnosis.

The remaining sections of this paper are structured as follows. The relevant theories and methods are described in Section 2. The framework diagram of the proposed fault diagnosis method is presented in Section 3. In Section 4, experimental studies are conducted to verify the diagnostic efficiency of the proposed method and compare it with other methods. Finally, the conclusions of this study are drawn in Section 5.

## 2. Methodology

### 2.1. VMD-Based Signal Processing

The input signal f(t) is decomposed into *K* IMFs by VMD. It obtains the optimal solution to the variational problem by adaptively matching the optimal center frequencies and bandwidths of the IMFs [29]. It can be described as follows:

(1) $$\left\{\begin{array}{l} \underset{\left\{U_{i}\},\{\omega_{i}\right\}}{\min}\left\{\sum_{i=1}^{K}{\left\|\partial_{t}\left[\left(\delta (t)+\frac{j}{\pi t}\right)*U_{i}(t)\right]e^{-j\omega_{i}t}\right\|}_{2}^{2}\right\}\\ s.t.\sum_{i=1}^{K}U_{i}(t)=f(t) \end{array}\right.$$

where $U_{i}(t)$ denotes the IMFs, $\omega_{i}$ represents the center frequency, ∗ stands for the convolution operator, $\partial_{t}$ indicates the derivative of the function with respect to time, and δ(t) is the unit impulse function.

A quadratic penalty factor α and a Lagrangian multiplier η(t) are employed to solve Equation (1), converting the constrained variational problem into an unconstrained problem, as follows:

(2) $$L(U_{i},\omega_{i},\eta )=\alpha \sum_{i}{\left\|\partial_{t}\left[\left(\delta (t)+\frac{j}{\pi t}\right)*U_{i}(t)\right]e^{-j\omega_{i}t}\right\|}_{2}^{2}+{\left\|f(t)-\sum_{i}U_{i}(t)\right\|}_{2}^{2}+\langle \eta (t),f(t)-\sum_{i}U_{i}(t)\rangle$$

Equation (2) is solved iteratively, and the Fourier isometric transform is used to convert the time domain problem into a frequency domain problem. The iterative process can be expressed as follows:

(3) $$\left\{\begin{array}{c} U_{i}^{n+1}(\omega )=\frac{f(\omega )-\sum_{i\neq k}U_{i}(\omega )+\frac{\eta (\omega )}{2}}{1+2\alpha {\left(\omega -\omega_{k}\right)}^{2}}\\ \eta^{n+1}(\omega )=\eta^{n}(\omega )+\lambda \left[f(\omega )-\sum_{k}U_{k}^{n+1}(\omega )\right]\\ \omega_{i}^{n+1}=\frac{\int_{0}^{\infty }\omega {\left|U_{i}(\omega )\right|}^{2}d\omega }{\int_{0}^{\infty }{\left|U_{i}(\omega )\right|}^{2}d\omega } \end{array}\right.$$

where λ is the noise tolerance and $U_{i}(\omega )$, η(ω), and f(ω) correspond to the Fourier transforms of $U_{i}(t)$, η(t), and f(t), respectively.

### 2.2. Fusion Entropy

Entropy can effectively describe the changes and fluctuations of signals, and different types of entropy often have distinct focuses in their expressions. RCMSE mainly emphasizes the repetitiveness and randomness of signals [19]. RCMDE has high computational efficiency, is sensitive to changes in signal amplitude, and possesses strong noise suppression capability [20]. RCMFE utilizes fuzzy functions, enabling more delicate differentiation of similar patterns and being less sensitive to noise and interference [30]. RCMFDE can simultaneously capture the similarity of signal patterns and the complexity of amplitude distribution, featuring comprehensive characteristic information [31]. Therefore, in this paper, a fusion entropy is proposed that incorporates various refined composite multi-scale entropies, making full use of the signal characteristics reflected by each type of entropy as features for fault diagnosis. A fusion entropy that includes RCMSE, RCMDE, RCMFE, and RCMFDE is proposed in this paper to comprehensively describe the changes and fluctuations of signals from different perspectives. The calculation steps of the fusion entropy are as follows:

Step 1: For a given time series $x=\left\{x_{1},x_{2},\ldots ,x_{N}\right\}$, the scale factor is set as *τ* and the embedding dimension is set as *m*.

Step 2: The original time series is subjected to coarse graining processing to obtain new sequences as follows:

(4) $$\left\{\begin{array}{l} u_{a}^{\tau }=[u_{a,1}^{\tau },u_{a,2}^{\tau },\ldots u_{a,i}^{\tau }]\\ u_{a,i}^{\tau }=\frac{1}{\tau }\sum_{k=(i-1)\tau +a}^{i\tau +a-1}x_{k} \end{array}\right.\;where\;a=1,2,\ldots ,\tau \;and\;i=1,2,\ldots ,(L=\left\lfloor \frac{N}{\tau }\right\rfloor )$$

where $u^{\tau }$ denotes the sequence after coarse graining.

Step 3: RCMSE is calculated [19]. Reconstructing template vectors are built with coarse-grained sequences $u^{\tau }$ as follows:

(5) $$z_{a,i}^{\tau ,m}=[u_{a,i}^{\tau },u_{a,i+1}^{\tau },\ldots ,u_{a,i+m-1}^{\tau }],\;i=1,2,\ldots ,(L-m+1)$$

The distance between the two template vectors $z_{a,i}^{\tau ,m}$ and $z_{a,j}^{\tau ,m}$ is calculated as follows:

(6) $$d_{a,i,j}^{\tau ,m}={\left\|z_{a,i}^{\tau ,m}-z_{a,j}^{\tau ,m}\right\|}_{\infty },\;where\;i,j=1,2,\ldots ,(L-m+1)\;and\;i\neq j$$

If the distance between the two template vectors is less than the similarity capacity *r*, then these two template vectors form a matching pair in the *m* dimensional space. The total number of matching pairs for the *i*-th template vector is $n_{a,i}^{\tau ,m}$.

The probability that any two template vectors are within r is calculated as follows:

(7) $${\tilde{n}}_{a}^{\tau ,m}=\frac{1}{L-m+1}\sum_{i=1}^{L-m+1}\frac{n_{a,i}^{\tau ,m}}{L-m}$$

${\tilde{n}}_{a}^{\tau ,m+1}$ is also calculated according to Steps 1 to 4, and RCMSE is calculated as follows:

(8) $$RCMSE(x,m,r,\tau )=-\ln\left(\frac{\sum_{a=1}^{\tau }{\tilde{n}}_{a}^{\tau ,m+1}}{\sum_{a=1}^{\tau }{\tilde{n}}_{a}^{\tau ,m}}\right)$$

Step 4: RCMFE is calculated [21]. The sequence $u^{\tau }$ is mapped to a high-dimensional space as follows:

(9) $$\left\{\begin{array}{l} S_{a,i}^{\tau ,m}=[u_{a,i}^{\tau },u_{a,i+1}^{\tau },\ldots ,u_{a,i+m-1}^{\tau }]-{\bar{u}}_{a,i}^{\tau },\;i=1,2,\ldots ,(L-m+1)\\ {\bar{u}}_{a,i}^{\tau }=\frac{1}{m}\sum_{j=0}^{m-1}u_{a,i+j}^{\tau } \end{array}\right.$$

The similarity distance $sd_{a,i,j}^{\tau ,m}$ of $S_{a,i}^{\tau ,m}$ and $S_{a,j}^{\tau ,m}$ is calculated as follows:

(10) $$sd_{a,i,j}^{\tau ,m}[S_{a,i}^{\tau ,m},S_{a,j}^{\tau ,m}]=\underset{p=0,1,\cdot \cdot \cdot ,L-m}{\max}\left[\left|S_{a,i}^{\tau ,m}(i+p)-S_{a,j}^{\tau ,m}(j+p)\right|\right],\;where\;i,j=1,2,\ldots ,(L-m+1)\;and\;i\neq j$$

The fuzzy similarity is calculated through an exponential fuzzy membership function with gradient steepness *n* and bandwidth *r* as follows:

(11) $$A_{a,i,j}^{\tau ,m}(sd_{a,i,j}^{\tau ,m})=\exp\left[-{\left(\frac{sd_{a,i,j}^{\tau ,m}}{r}\right)}^{n}\right]$$

The similarity of high-dimensional space is calculated as follows:

(12) $$\phi_{a}^{\tau ,m}(n,r)=\frac{1}{L-m}\sum_{i=1}^{L-m}\left(\frac{1}{L-m-1}\sum_{j=1,j\neq i}^{L-m}A_{a,i,j}^{\tau ,m}\right)$$

Then, RCMFE is calculated as follows:

(13) $$\left\{\begin{array}{l} RCMFE(x,m,n,r,\tau )=-\ln\left(\frac{{\bar{\phi }}^{\tau ,m+1}}{{\bar{\phi }}^{\tau ,m}}\right)\\ {\bar{\phi }}^{\tau ,m}=\frac{1}{\tau }\sum_{a=1}^{\tau }\phi_{a}^{\tau ,m}(n,r) \end{array}\right.$$

Step 5: RCMDE is calculated [20]. The coarse-grained sequences $u^{\tau }$ are mapped to *c* classes, with integer indices ranging from 1 to *c*, as follows:

(14) $$\left\{\begin{array}{l} y_{a,i}^{\tau }=\frac{1}{\sigma \sqrt{2\pi }}\int_{-\infty }^{u_{a,i}^{\tau }}e^{\frac{-{\left(t-mv\right)}^{2}}{2\sigma^{2}}}dt\\ dz_{a,i}^{\tau }=round(c\cdot y_{a,i}^{\tau }+0.5) \end{array}\right.$$

where σ and mv are the standard deviation and mean value of $u_{a}^{\tau }$, round(⋅) represents the rounding function, and $dz_{a,i}^{\tau }$ is the class corresponding to $u_{a,i}^{\tau }$.

Time series $ze_{a,j}^{m,\tau ,c}$ is established with an embedding dimension m and a time delay d as follows:

(15) $$ze_{a,j}^{m,\tau ,c}=\{dz_{a,j}^{\tau ,c},dz_{a,j+d}^{\tau ,c},dz_{a,j+2d}^{\tau ,c}\ldots ,dz_{a,j+(m-1)d}^{\tau ,c}\},\;\;j=1,2,\cdot \cdot \cdot ,L-(m+1)d$$

Each $ze_{a,j}^{m,\tau ,c}$ is mapped to a dispersion pattern $\pi_{v_{0}v_{1}\cdot \cdot \cdot v_{m-1}}$, where $de_{a,j}^{\tau ,c}=v_{0}$, $dz_{a,j+d}^{\tau ,c}=v_{1}$,…,$dz_{a,j+(m-1)d}^{\tau ,c}=v_{m-1}$. The relative frequency rf of each dispersion pattern is calculated as follows:

(16) $$rf(\pi_{v_{0}v_{1}\cdot \cdot \cdot v_{m-1}},ze_{a,j}^{m,\tau ,c})=\frac{Number(\pi_{v_{0}v_{1}\cdot \cdot \cdot v_{m-1}}|ze_{a,j}^{m,\tau ,c})}{L-(m-1)d}$$

where $Number(\pi_{v_{0}v_{1}\cdot \cdot \cdot v_{m-1}}|z_{a,j}^{m,\tau ,c})$ represents the number of patterns $\pi_{v_{0}v_{1}\cdot \cdot \cdot v_{m-1}}$ that appear in the time series $ze_{a,j}^{m,\tau ,c}$.

RCMDE can be calculated as follows:

(17) $$RCMDE(x,\tau ,m,c,d)=-\sum_{\pi =1}^{c^{m}}(\frac{1}{\tau }\sum_{a=1}^{\tau }rf(\pi_{v_{0}v_{1}\cdot \cdot \cdot v_{m-1}},ze_{a,j}^{m,\tau ,c})).\ln(\frac{1}{\tau }\sum_{a=1}^{\tau }rf(\pi_{v_{0}v_{1}\cdot \cdot \cdot v_{m-1}},ze_{a,j}^{m,\tau ,c}))$$

Step 6: RCMFDE is calculated [22]. The coarse-grained sequences $u^{\tau }$ are mapped to *c* classes as shown in Equation (14). The degree of membership between each $dz_{a,i}^{\tau }$ to the *k*th class (*k* = 1, …, c) is presented according to a fuzzy membership as follows:

(18) $$\mu_{M_{k}}(dz_{a,i}^{\tau ,c})=\left\{\begin{array}{l} \left\{\begin{array}{l} 0\;\;\;\;\;\;\;dz_{a,i}^{\tau ,c}>2\\ 2-dz_{a,i}^{\tau ,c}\;\;\;\;1\leq dz_{a,i}^{\tau ,c}\leq 2\\ 1\;\;\;\;\;\;\;dz_{a,i}^{\tau ,c}<1 \end{array}\right.\;\;\;when\;k=1\\ \left\{\begin{array}{l} 0\;\;\;\;\;\;\;dz_{a,i}^{\tau ,c}>k+1\\ k+1-dz_{a,i}^{\tau ,c}\;\;k\leq dz_{a,i}^{\tau ,c}\leq k+1\\ dz_{a,i}^{\tau ,c}-k+1\;\;k-1\leq dz_{a,i}^{\tau ,c}<k\\ 0\;\;\;\;\;\;\;dz_{a,i}^{\tau ,c}<k-1 \end{array}\right.\;when\;k=2,\ldots ,c-1\\ \left\{\begin{array}{l} 1\;\;\;\;\;\;\;dz_{a,i}^{\tau ,c}>c\\ dz_{a,i}^{\tau ,c}-c+1\;\;c-1\leq dz_{a,i}^{\tau ,c}\leq c\\ 0\;\;\;\;\;\;\;dz_{a,i}^{\tau ,c}<c-1 \end{array}\right.\;\;when\;k=c \end{array}\right.$$

Time series $ze_{a,j}^{m,\tau ,c}$ is established with an embedding dimension m and a time delay d according to Equation (15). The probability of each dispersion pattern is calculated as follows:

(19) $$p_{a}^{\tau }(\pi_{v_{0}v_{1}\cdot \cdot \cdot v_{m-1}})=\frac{\sum_{j=1}^{L-(m-1)d}\left(\prod_{i=0}^{m-1}\mu_{Mv_{i}}(dz_{a,j+(i)d}^{\tau ,c})\right)}{L-(m-1)d}$$

RCMFDE can be calculated as follows:

(20) $$RCMFDE(x,\tau ,m,c,d)=-\sum_{\pi =1}^{c^{m}}(\frac{1}{\tau }\sum_{a=1}^{\tau }p_{a}^{\tau }(\pi_{v_{0}v_{1}\cdot \cdot \cdot v_{m-1}})).\ln(\frac{1}{\tau }\sum_{a=1}^{\tau }p_{a}^{\tau }(\pi_{v_{0}v_{1}\cdot \cdot \cdot v_{m-1}}))$$

Step 7: The fusion entropy, which serves as the feature vector, is collectively composed of RCMSE, RCMFE, RCMDE, and RCMFDE:

(21) Fusionentropy(x,τ,m,c,d,r,n)=[RCMSE,RCMDE,RCMFE,RCMFDE]

### 2.3. Feature Selection Utilizing RF

Although various advantages of different types of entropies have been utilized in the fusion entropy, these entropies still exhibit great similarities. Therefore, the fusion entropy also contains a large amount of redundant and interfering information in the multi-scale entropy and fusion entropy, which may sometimes interfere with accurate judgment in fault diagnosis. Therefore, it is necessary to perform feature selection. RF can analyze the importance of each feature to select those that have a greater impact on fault diagnosis [32], thereby improving the efficiency of fault diagnosis. The steps for calculating the feature importance of RF are as follows.

Assume that the number of samples in the dataset used for diagnosis is *M*. From the dataset, g samples (g < M)) are randomly selected with replacement to form the in-bag dataset, while the remaining unselected samples constitute the out-of-bag (OOB) dataset, which is used as the test set.

The RF model is trained using the in-bag dataset, and voting is performed using the OOB dataset. By randomly altering the values of the *P*-th feature in the OOB dataset and re-conducting the voting, the calculation process for the importance of the *P*-th feature $IMP_{P}$ is as follows:

(22) $$IMP_{P}=\frac{1}{N_{tree}}\sum_{i=1}^{N_{tree}}\left(SK_{P}-SX_{P}\right)$$

where $N_{tree}$ is the number of decision trees and $SK_{P}$ and $SX_{P}$ are the voting scores of the *i*-th decision tree on the corresponding OOB dataset before and after randomly altering the *P*-th feature, respectively. By eliminating features with lower importance, the computational complexity of the fault diagnosis model can be reduced, while unnecessary interference can be eliminated.

### 2.4. LSTM

**Figure 1:**
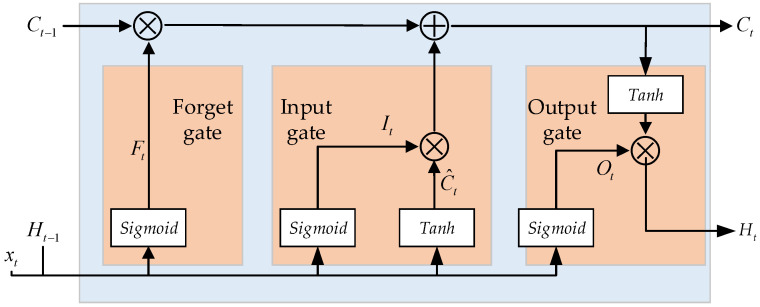
The structure diagram of the LSTM memory cell.

LSTM is an improved form of a recurrent neural network (RNN). It enables stable transmission of information in long sequences through memory cells and gating mechanisms, thereby achieving efficient prediction and classification [33]. The structure diagram of the LSTM memory cell is shown in Figure 1, where $C_{t-1}$ and $H_{t-1}$ represent the memory cell state and hidden layer state at the previous moment, respectively, and $C_{t}$ and $H_{t}$ denote the memory cell state and hidden layer state at the current moment, respectively. $F_{t}$, $I_{t}$, and $O_{t}$ stand for the gating states of the forget gate, input gate, and output gate, respectively. *Sigmoid* and *tanh* represent the Sigmoid activation function and hyperbolic tangent activation function, respectively, while ⊕ and ⊗ denote the matrix summation operator and Hadamard product operator, respectively.

Within a computational time step corresponding to time *t*, the input $x_{t}$ will sequentially pass through the forget gate, input gate, and output gate. Finally, the output gate determines the output $H_{t}$ at the current time, while updating the state $C_{t}$ of the memory cell. The specific calculation process is as follows:

Step 1: The gating state $F_{t}$ of the forget gate at time *t* is computed to determine whether historical information should be retained or discarded as follows:

(23) $$F_{t}=Sigmoid(W_{f}[x_{t},H_{t-1}]+b_{f})$$

where $W_{f}$ and $b_{f}$ represent the weight matrix and bias vector of the gating state of the forget gate, respectively. A value of 0 for $F_{t}$ indicates discarding all historical information, while a value of 1 for $F_{t}$ indicates retaining all information.

Step 2: The gating state $I_{t}$ and candidate state ${\hat{C}}_{t}$ of the input gate are calculated as follows:

(24) $$I_{t}=Sigmoid(W_{i}[x_{t},hd_{t}-1]+b_{i})$$

(25) $${\hat{C}}_{t}=\tanh(W_{c}[x_{t},hd_{t-1}]+b_{c})$$

where $W_{i}$ and $W_{c}$ represent the weight matrices for the gating state and candidate state of the input gate, respectively. $b_{i}$ and $b_{c}$ denote the bias vectors for the gating state and candidate state of the input gate, respectively.

Step 3: The state $C_{t}$ of the memory cell at time *t* is updated as follows:

(26) $$C_{t}=F_{t}⊗C_{t-1}+I_{t}⊗{\hat{C}}_{t}$$

Step 4: The gating state $O_{t}$ of the output gate and the hidden state $H_{t}$ are updated to obtain the output of the LSTM memory cell at time *t* as follows:

(27) $$O_{t}=Sigmoid(W_{o}[x_{t},hd_{t-1}]+b_{o})$$

(28) $$H_{t}=O_{t}⊗\tanh(C_{t})$$

where $W_{o}$ and $b_{o}$ represent the weight matrix and bias vector of the gating state of the output gate, respectively.

## 3. The Structure of the Proposed Method

**Figure 2:**
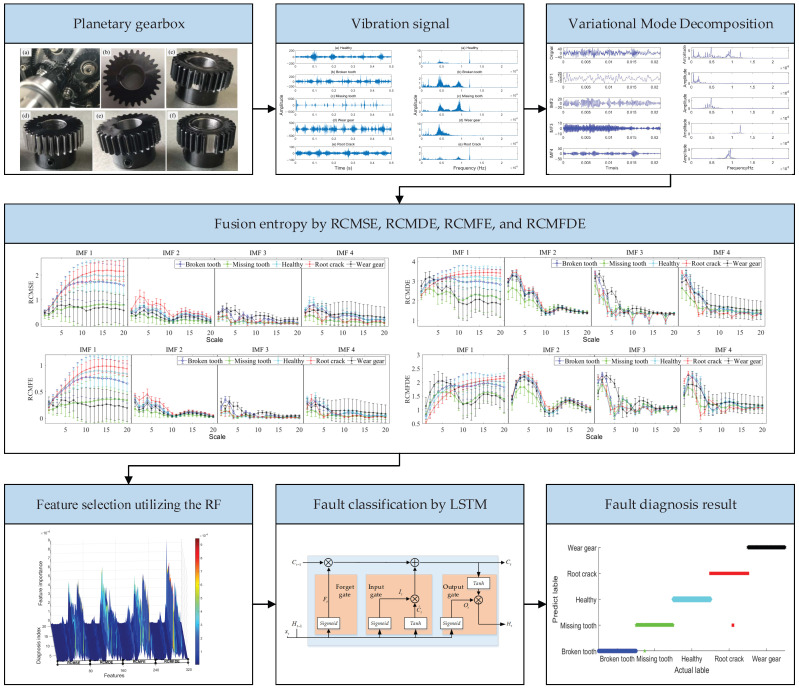
The framework diagram of the proposed fault diagnosis method.

In this paper, a hybrid fault diagnosis method based on VMD signal processing, fusion entropy feature extraction, RF feature selection, and LSTM classification is proposed for planetary gearboxes. To more clearly demonstrate the specific processes of data processing, feature extraction, and fault classification, the framework diagram of the proposed method is illustrated in Figure 2 [34]. The main steps of the method proposed in this paper are as follows.

The main steps of the fault diagnosis method proposed in this paper are as follows:

Step 1: The vibration data of the planetary gearbox under different fault and operating conditions is acquired through data collection devices.

Step 2: The time domain vibration signal is decomposed into IMFs with different frequencies using the VMD algorithm, thereby separating vibrations and trends of different scales in the original vibration signal.

Step 3: The fusion entropy of each IMF is calculated, which includes RCMSE, RCMDE, RCMFE, and RCMFDE. The fusion entropy fully utilizes the signal characteristics reflected by various entropies as features for fault diagnosis.

Step 4: The feature is selected by RF based on feature importance. Although the fusion entropy incorporates the advantages of various refined composite multi-scale entropies, there exists a large amount of redundancy in the features, and a large number of noise entropy values may appear in the calculation of multi-scale entropy values, which affect the accuracy and efficiency of fault classification. Using RF to evaluate the importance of feature entropy values and selecting features with higher importance to form a new feature set, the efficiency of fault diagnosis can be effectively improved.

Step 5: The new feature set obtained in Step 4 is divided into a training set and a test set. The parameters of the long short-term memory (LSTM) network are initialized, and the LSTM network is trained using the training set until the target accuracy is achieved.

Step 6: Then, the test set is input into the LSTM for testing. The fault diagnosis accuracy of the test set is used as an indicator to evaluate the diagnostic efficiency.

## 4. Experimental Study

### 4.1. Experimental Description

**Figure 3:**
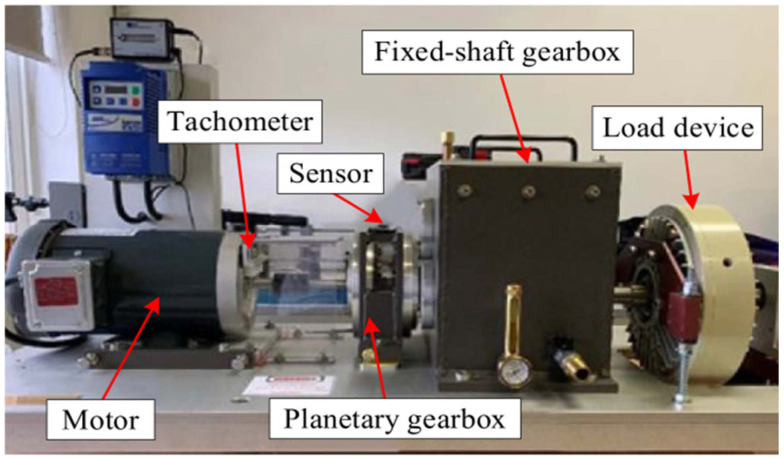
Experimental setup [34,35].

**Figure 4:**
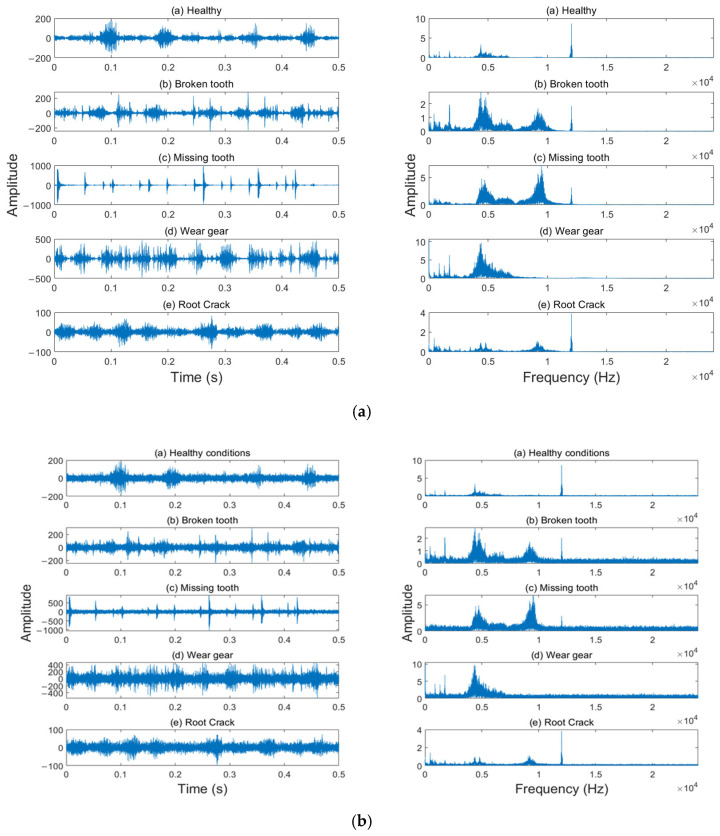
The time and frequency domain waveforms of the vibration signals. (**a**) Original vibration data; (**b**) with additional white noise; SNR = 3 dB.

In this experiment, the vibration data of the planetary gearbox under various operating conditions were publicly provided by Liu et al. [35]. The data can be obtained from the following link: https://github.com/Liudd-BJUT/WT-planetary-gearbox-dataset (accessed on 5 November 2024). This dataset was constructed by collecting vibration data of the planetary gearbox under different operating states on an experimental test platform, as shown in Figure 3 [34,35]. The main parameters of the planetary gearbox and the data collection device are listed in Table 1. The planetary gearbox includes five different states, namely, the healthy state, broken tooth, missing tooth, gear wear, and root crack of the sun gear. The time domain and frequency domain waveforms of the originally collected vibration signals are shown in Figure 4a. To verify the fault diagnosis efficiency of the method proposed in this paper in a noisy environment, white noise with a signal-to-noise ratio (SNR) of 3 dB was additionally added to the originally collected vibration signals, and the time domain and frequency domain waveforms of the signals are shown in Figure 4b.

As can be observed from Figure 4, regarding the time domain waveforms, although different types of faults exhibit distinctions on a larger time scale, it is rather difficult to differentiate between various fault types relying solely on the time domain waveforms within a shorter time window. In the frequency domain, distinguishing among different fault types is equally challenging. In this study, short data samples are employed, effective features are selected through the proposed fault feature extraction and selection method, and LSTM is adopted as the fault classifier.

The data of each operating state in the original dataset is first divided into 400 sample data segments of equal length, and 1024 consecutive vibration data points are randomly selected from each segment to form the sample set. Among them, 60% of the samples under each operating state are randomly chosen as the training set, and the remaining 40% serve as the test set. The specific division parameters and label settings of the sample set are listed in Table 2.

The experimental process described in Section 4 was performed with MATLAB 2022b on a computer (Lenovo made in Beijing, China) equipped with an Intel i7-8700 CPU (3.2 GHz) and 16 GB of RAM in Suqian, China.

### 4.2. Signal Processing

**Figure 5:**
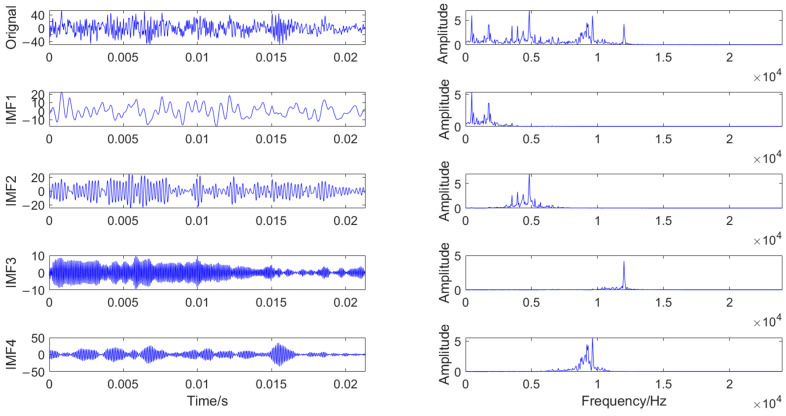
The time domain and frequency domain diagrams of the original vibration signal and the decomposed IMFs under the broken tooth state.

VMD was employed to decompose the vibration signals, aiming to extract vibrations and trends at different scales embedded within the signals. Among the parameters of VMD, the modal parameters *K* and *α* require manual setting. Based on the frequency domain characteristics of vibration signals under different operating states, as shown in Figure 4, it can be observed that their features are mainly concentrated within three or four frequency ranges. Therefore, in this study, the decomposition modal parameter *K* of VMD was set to 4, and the decomposition parameter *α* was selected as 500. Taking the vibration signal under the broken tooth state as an example for decomposition, the time domain and frequency domain diagrams of the original vibration signal and the decomposed IMFs are presented in Figure 5.

As can be seen from Figure 5, after VMD decomposition, the signal is decomposed into IMF components within different frequency ranges. These IMF components exhibit more regular patterns, which facilitates the extraction of fault features.

### 4.3. Feature Extraction and Fusion

The fusion entropy is proposed as the feature for fault diagnosis in this paper. To verify that different types of entropy values in the fusion entropy, as fault features, can complement each other and improve the efficiency of fault diagnosis, RCMSE, RCMDE, RCMFE, and RCMFDE were calculated for 400 samples under each operating state, respectively. The calculation parameters of different entropy values are listed in Table 3. The software for the calculation of entropy value in MATLAB 2018a can be obtained from the following link: https://github.com/HamedAzami/NLDyn/blob/main/Final_V10p.rar (accessed on 24 December 2024).

**Figure 6:**
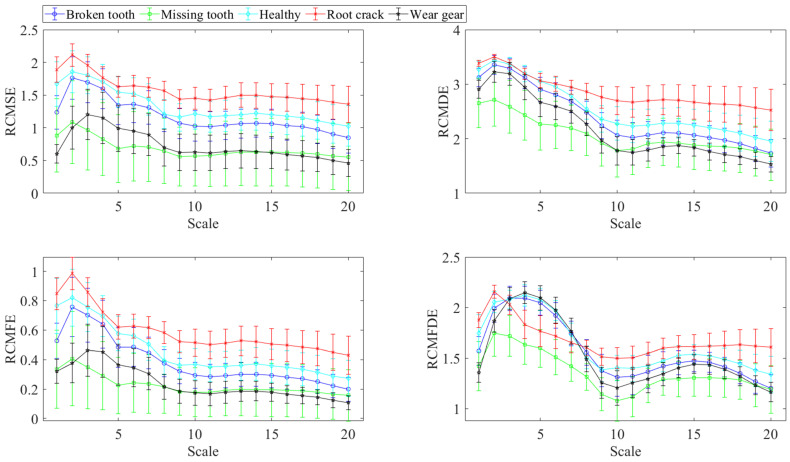
The mean values and SDs by different entropies of the original sample.

**Figure 7:**
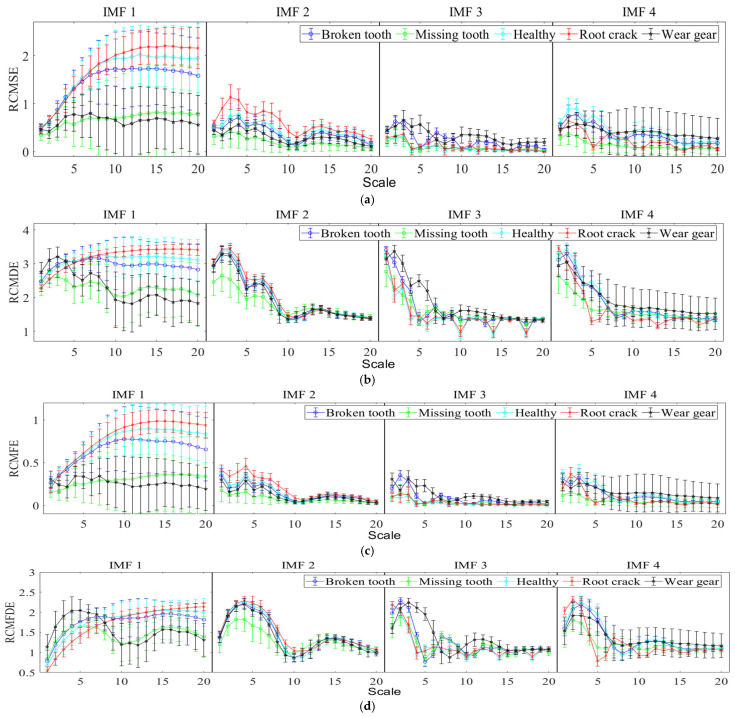
The mean values and SDs by different entropies of the sample signal after VMD decomposition. (**a**) RCMSE; (**b**) RCMDE; (**c**) RCMFE; and (**d**) RCMFDE.

Meanwhile, to further verify the role of VMD decomposition, a comparison was made between the entropy values of the original sample signals and those of the decomposed samples. Figure 6 presents the mean values and standard deviations (SDs) of various multi-scale refined composite entropy values of the original signals. Figure 7 shows the mean values and SDs of various multi-scale refined composite entropy values of each IMF obtained after decomposing the original signals.

As can be observed from Figure 6 and Figure 7, the discriminability among various operating states has been enhanced after VMD. For example, in the fifth scale of IMF3, the wear gear state can be clearly distinguished from several other states. Additionally, in the second scale of RCMFE corresponding to IMF3, the broken tooth state also exhibits a favorable level of discriminability. These results indicate that the feature extraction of the signal has been strengthened through VMD decomposition.

**Figure 8:**
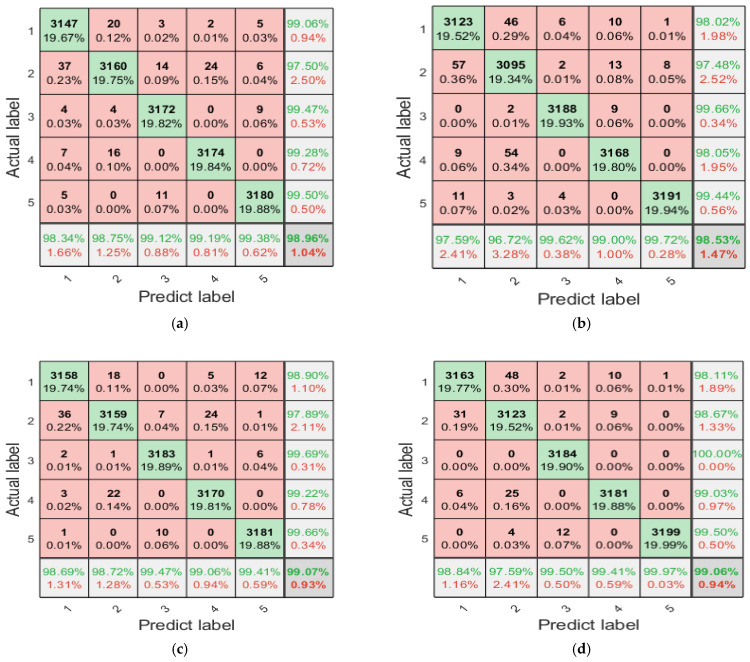
Confusion matrix of diagnosis using individual entropy values with the original sample set (summary of 20 runs). (**a**) RCMSE; (**b**) RCMDE; (**c**) RCMFE; and (**d**) RCMFDE.

**Figure 9:**
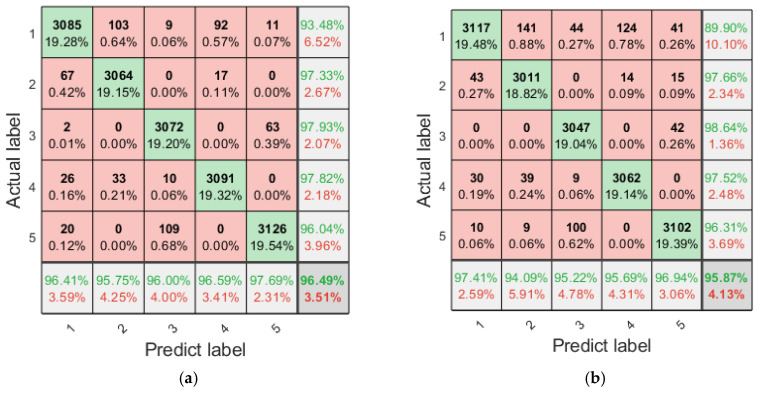

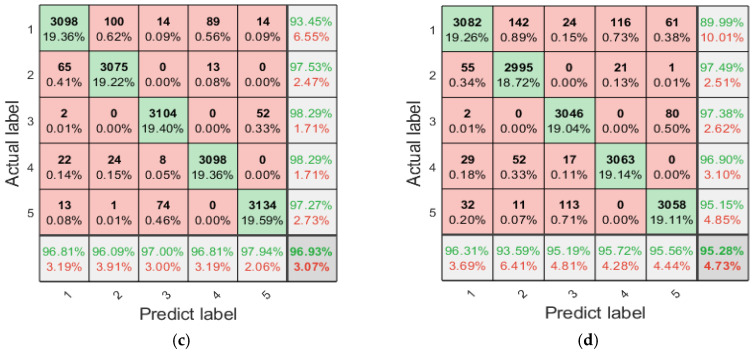
Confusion matrix of diagnosis using individual entropy values with additional white noise; SNR = 3 dB (summary of 20 runs). (**a**) RCMSE; (**b**) RCMDE; (**c**) RCMFE; and (**d**) RCMFDE.

The fault classification using multi-scale entropy necessitates a comprehensive consideration of the performance of entropy values across multiple scales under different states; it is challenging to intuitively discern the classification efficiency of different entropy features. To address this issue, LSTM is employed for classification, with an analysis conducted on the fault diagnosis efficiency when each single multi-scale refined composite entropy is used as a feature. The training and test sets of LSTM are configured as specified in Table 2. The LSTM model is structured with 128 hidden layer units, 20 input layer units, and five output layer units, and it is trained for 1000 epochs. To mitigate the impact of randomness, each fault diagnosis method is executed independently 20 times, which entails that the training and test sets are also configured independently 20 times. The 20 fault diagnosis results obtained using the single multi-scale refined composite entropy as the feature are aggregated, and the confusion matrix of the diagnosis results is presented in Figure 8 and Figure 9.

As can be seen from Figure 8, RCMFED achieves the best classification performance on the first, fourth, and fifth categories; RCMDE performs best on the third category; and both RCMSE and RCMFE show relatively good classification results on the second category. In terms of overall accuracy, RCMFDE and RCMFE yield better performance. It can be observed from Figure 9 that with the additional white noise, RCMFE exhibits the best classification effect on the second, third, fourth, and fifth categories, while RCMDE performs optimally on the first category. In terms of overall accuracy, RCMFE achieves the best result, indicating that RCMFE has a strong ability to resist noise.

**Figure 10:**
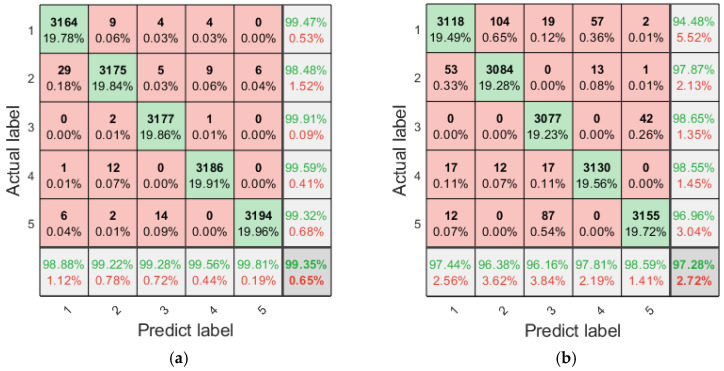
Confusion matrix of diagnosis using fusion entropy (summary of 20 runs). (**a**) Original signal; (**b**) with additional white noise; SNR = 3 dB.

The fault diagnosis results of the aforementioned single multi-scale refined composite entropy values indicate that different entropy values exhibit varying efficiencies in diagnosing different types of faults, with each entropy value possessing certain advantages. To address this, this paper proposes the use of fusion entropy as the feature for planetary gearbox fault diagnosis. Similarly, with fusion entropy serving as the input to the LSTM, 20 independent fault diagnosis runs are conducted, and the confusion matrix summarizing the diagnosis results is presented in Figure 10.

The overall accuracy of fault diagnosis with fusion entropy has been improved compared with that of diagnosis using a single entropy value. However, the diagnosis accuracy rate of some types of faults has decreased. The reason for this phenomenon is that after feature fusion, there is a large amount of information redundancy and interference in the feature samples, which affects the efficiency of fault diagnosis and classification.

### 4.4. Feature Selection

**Figure 11:**
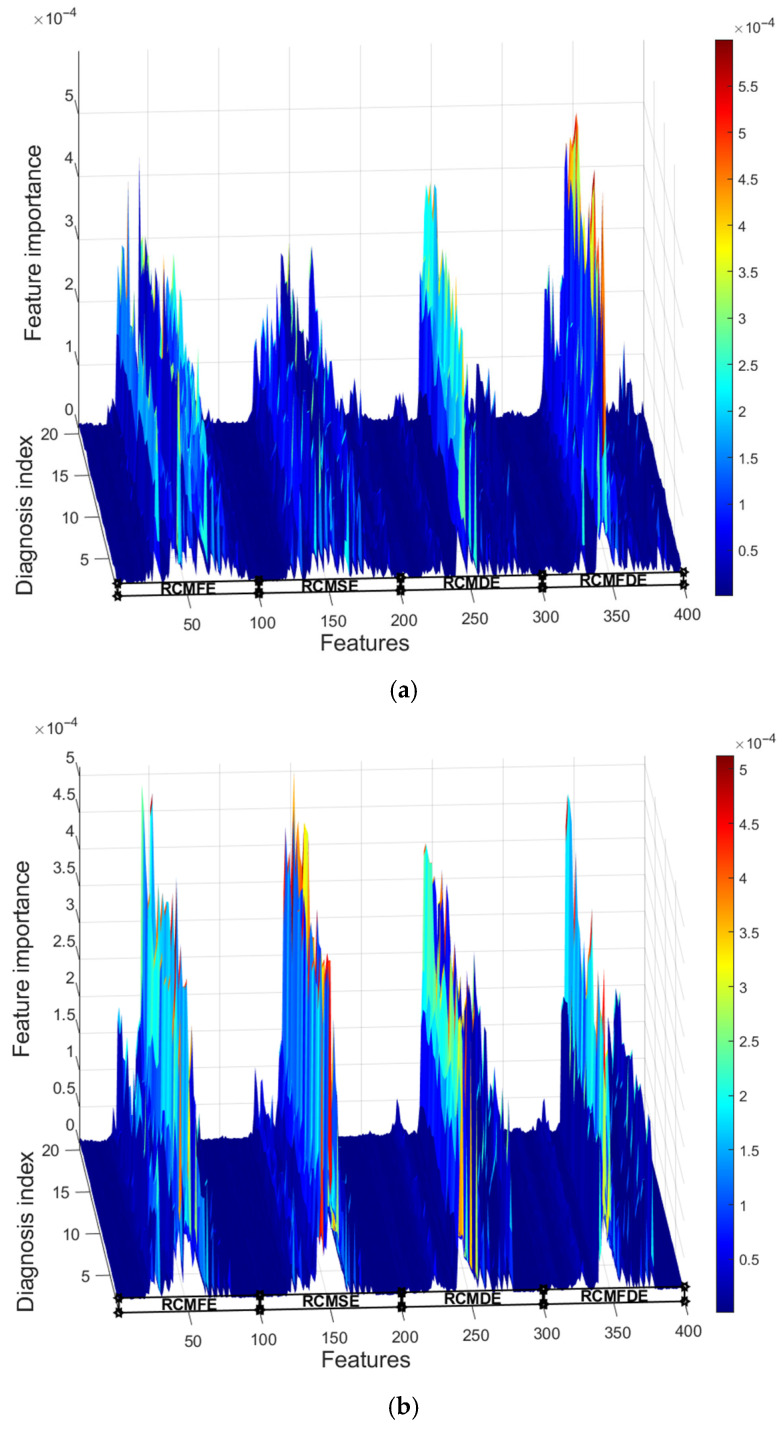
Waterfall plot of feature importance. (**a**) Original signal; (**b**) with additional white noise; SNR = 3 dB.

To overcome the interference of unnecessary feature information and further improve the efficiency of fault diagnosis, RF is employed for feature selection. A total of 60% of the fusion entropy feature set is randomly selected as the training set to be input into the RF model, with the number of decision trees set to 1000. The feature set input to RF is independently selected 20 times, and the results of the feature importance IMP evaluation by the RF model are shown in Figure 11.

As can be observed from Figure 11, there are significant differences in the importance of different features, indicating the need to eliminate redundant features and select those with higher importance. Meanwhile, Figure 11 also shows that although the datasets for the 20 independent tests are different, the distinction in importance among features maintains a high level of consistency and does not vary significantly due to the difference in dataset selection. The results indicate that the proposed method has good generalization ability.

**Figure 12:**
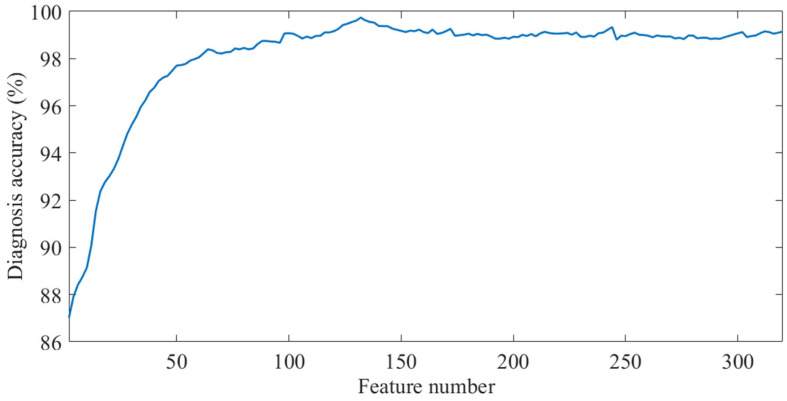
Impact of selecting different numbers of features on diagnostic accuracy.

Features are sorted and selected in descending order of their importance, and LSTM is utilized for fault diagnosis. Figure 12 illustrates the impact of selecting different numbers of features on diagnostic accuracy.

**Figure 13:**
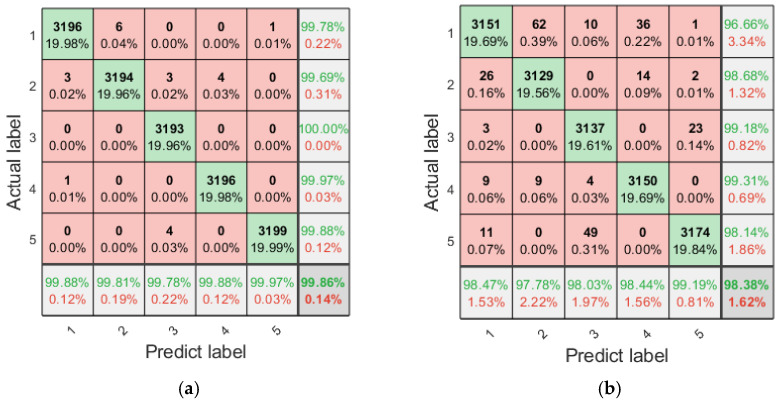
Confusion matrix of the diagnosis results of the proposed method (summary of 20 runs). (**a**) Original signal; (**b**) with additional white noise; SNR = 3 dB.

As can be seen from Figure 12, when the number of input features is small, LSTM fails to obtain sufficient feature information for fault classification, resulting in low accuracy. With the gradual increase in the number of input features, the accuracy also increases gradually, reaching a peak when the number of input features reaches 132. As the number of features continues to increase, the accuracy begins to decrease and gradually stabilizes, indicating that redundant feature information has a certain negative impact on the classification performance of the model. The top 132 features with higher importance are selected as the input features of LSTM, and 20 independent fault diagnosis runs are conducted. The confusion matrix summarizing the diagnosis results is presented in Figure 13.

A comparison between Figure 10 and Figure 13 reveals that after feature selection using RF, the classification efficiency of LSTM has been improved. The fault diagnosis accuracy for the original signal reaches 99.86%, and even under the condition of additional 3dB noise, the fault diagnosis accuracy still reaches 98.38%.

### 4.5. Fault Diagnosis Analysis

Since this study adopts the fusion of multiple signal processing methods, feature extraction methods, and feature selection methods for fault diagnosis, ablation experiments are conducted to verify the roles of VMD, fusion entropy, and RF-based feature selection in the entire diagnostic framework. The results of the ablation experiments are presented in Table 4.

From the results of the ablation experiments, it can be observed that VMD exerts a significant effect on improving the fault diagnosis accuracy. Specifically, the proposed fusion entropy in this study achieves higher accuracy compared with a single entropy value. Additionally, RF-based feature selection also contributes to enhancing the fault diagnosis accuracy.

### 4.6. Fault Diagnosis Under Different Rotational Speeds

**Figure 14:**
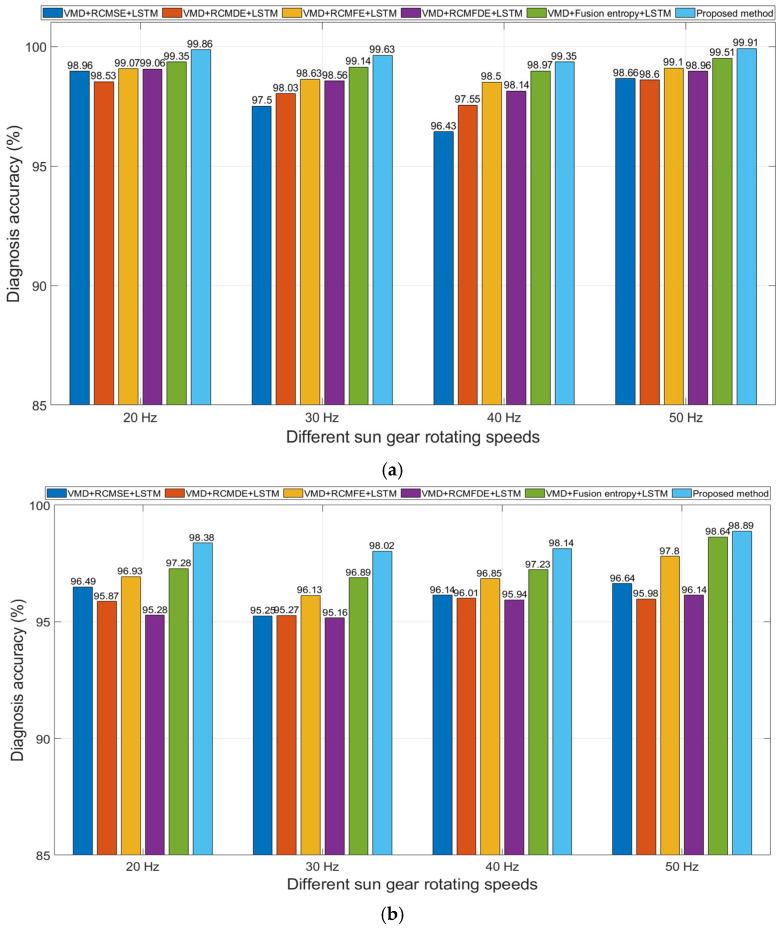
Fault diagnosis accuracy of different methods under different sun gear rotational speeds. (**a**) Original signal; (**b**) with additional white noise; SNR = 3 dB.

To verify the fault diagnosis capability of the proposed method in this paper under different sun gear rotational speeds, vibration data under sun gear rotational frequencies of 20 Hz, 30 Hz, 40 Hz, and 50 Hz were subjected to fault diagnosis. Among them, features based on single entropy values (RCMSE, RCMFE, RCMDE, and RCMFDE) and features based on fusion entropy values were also used for fault classification to be compared with the proposed method. The training sample sets and test sets of the data were independently generated 20 times, and the average accuracy rates of 20 fault diagnosis runs based on various features are shown in Figure 14.

The results presented in Figure 14 indicate that the fusion entropy achieves higher fault diagnosis accuracy than single entropy values under various conditions, demonstrating that the fusion entropy proposed in this paper can more comprehensively reflect the operating status of the planetary gearbox. After feature selection using RF, the efficiency of fault diagnosis is further improved. Without additional noise, the average fault diagnosis accuracy of the proposed method at each rotational speed exceeds 99.35%; with the addition of 3dB extra white noise, the average fault diagnosis accuracy of the proposed method at each rotational speed still exceeds 98.02%. These results indicate that there are some redundant features in the fused entropy that have a negative impact on fault classification, and RF-based feature selection can effectively eliminate such redundant features and improve the efficiency of fault diagnosis. Overall, the results demonstrate that the proposed method is applicable to fault diagnosis of planetary gearboxes under different sun gear rotational speeds and exhibits good noise suppression capability.

## 5. Conclusions

Extracting effective fault features from the complex vibration signals of planetary gearboxes is the key to conducting efficient fault diagnosis, and it involves signal processing, feature extraction, and feature selection. This paper proposes a novel fault feature extraction method based on VMD, fusion entropy, and feature selection by RF. Meanwhile, LSTM is also utilized to classify the feature vectors constructed by the proposed method for fault diagnosis. The following conclusions are drawn from the experimental studies:

(1) The experimental results show that feature extraction methods based on various entropy values differ in the aspects of signals they reflect. Different entropy values, when used as fault features, exhibit varying efficiencies in diagnosing different types of faults, with each entropy value having certain advantages. The use of fusion entropy as a fault feature can effectively improve the accuracy of fault diagnosis.
(2) There exists a large number of redundant features in fusion entropy. Through the evaluation of feature importance by RF, it is found that there are significant differences in the importance of different features. Features with higher importance are selected to reconstruct the fault diagnosis feature vector.
(3) The feature vectors processed by VMD, fusion entropy, and RF feature selection are used as inputs to the LSTM classifier for fault diagnosis of planetary gearboxes. The diagnosis results indicate that the proposed method has good diagnostic capability for planetary gearbox and also excellent performance and noise suppression capability.

For future studies, to address the training bottleneck caused by data silos in industrial scenarios, we plan to integrate the FedProx algorithm with the feature extraction method presented in this paper. The combined approach will be used to alleviate parameter drift in the long short-term memory (LSTM) model under distributed conditions, with the ultimate goal of realizing privacy-preserving fault diagnosis across clients from different wind farms.

## Figures and Tables

**Table 1:** The parameters of the experimental setup.

Parameter Description		Values
Tooth number	Sun gear	28
	Ring gear	100
	Planet gear (number)	36 (4)
Sun gear rotating frequency		20 Hz, 30 Hz, 40 Hz, 50 Hz
Sampling frequency		48,000 Hz

**Table 2:** Parameters of the sample dataset.

Operation State	Sample Length	Number of Samples	The Division Ratio of the Training Set and Test Set	Operation Label
Healthy	1024 points	400	6:4 (The samples were randomly selected)	1
Broken tooth		400		2
Missing tooth		400		3
Root crack		400		4
Wear gear		400		5

**Table 3:** Parameter settings for entropy calculation methods.

Parameter Description	Values
Scale factors, τ	20
Scalar embedding, m	2
Scalar time lag, d	1
Number of classes, c	6
Scalar threshold, r	0.15
Fuzzy power, n	2

**Table 4:** The results of the ablation experiments on fault diagnosis.

Methods	Fault Diagnosis Accuracy (Original Signal)	Fault Diagnosis Accuracy (SNR = 3 dB)
Fusion entropy + RF + LSTM	98.15%	92.42%
VMD + RCMSE + RF + LSTM	99.07%	97.18%
VMD + RCMFE+ RF + LSTM	98.93%	96.81%
VMD + RCMDE + RF + LSTM	99.37%	97.58%
VMD + RCMFDE + RF + LSTM	99.31%	96.31%
VMD + fusion entropy + LSTM	99.35%	97.28%
VMD + fusion entropy + RF + LSTM	99.86%	98.38%

## Data Availability

The data that support the findings of this study are available upon reasonable request from the authors.
